# Penetration and Development of *Meloidogyne Javanica* on Four Pistachio Rootstocks and their Defense Responses

**DOI:** 10.2478/jofnem-2022-0056

**Published:** 2023-01-20

**Authors:** Fatemeh Shekari Mahoonaki, Esmat Mahdikhani Moghadam, Mohammad Zakiaghl, Majid Pedram

**Affiliations:** 1 Department of Plant Protection, Faculty of Agriculture, Ferdowsi University of Mashhad, Mashhad, Iran; 2 Department of Plant Pathology, Faculty of Agriculture, Tarbiat Modares University, Tehran, Iran

**Keywords:** development, hypersensitive response, penetration, pistachio, resistance, root-knot nematode

## Abstract

Pistachio yield is annually reduced due to root-knot nematode infections. In order to evaluate its resistance to *Meloidogyne javanica*, three domestic pistachio rootstocks, namely, Badami, Ghazvini and Sarakhs, and a wild pistachio, Baneh (*Pistacia atlantica* subsp. *mutica*), were selected. Their response to the nematode infection was evaluated based on different plant and nematode indexes, 120 days post-inoculation (dpi). The penetration and development rate of nematode in roots of these four pistachio rootstocks were evaluated at different time points by acid fuchsin staining. Based on the measured indexes, Badami, Ghazvini, Sarakhs, and Baneh rootstocks ranked as susceptible, moderately resistant, moderately resistant, and resistant, respectively. The penetration rate of second-stage nematode juveniles (J2) into four rootstocks was discussed. The first “midstage” or swollen juveniles appeared at 4 dpi but to a lesser extent in Ghazvini, Sarakhs, and Baneh cultivars. The first females were seen in Badami at 21 dpi, in Ghazvini and Sarakhs at 35 dpi, and in Baneh at 45 dpi. Three types of defense responses were distinguished and characterized in the examined pistachio rootstocks: (i) a hypersensitive response (HR)-like reaction in the cortex in Ghazvini, Sarakhs, and Baneh root tips at 4 dpi and 6 dpi; (ii) an HR response, degrading J2 which induce giant cells in the vascular cylinder of all rootstocks, at 6 dpi and 10 dpi; and (iii) an HR response, degrading females and giant cells in the vascular cylinder of all rootstocks at 15 dpi onward. These observations open new fields of study in breeding programs of this crop.

Root-knot nematodes, *Meloidogyne* spp., are one of the most important pathogens of pistachio trees, dominating most pistachio-growing areas in Iran. They are sedentary obligate endoparasitic nematodes with a wide host range and can remarkably damage and reduce yield production. The second-stage juveniles (J2) nematode usually enter roots near the tip, behind the cap, and moves intercellularly between cortical cells downward to the root tip and enter the vascular cylinder. About five to seven parenchymatic root cells around the nematode head grow and form giant cells, after injecting the nematode effectors, triggering the reprogramming of the target cells. *Meloidogyne* spp. disrupt host physiology and reduce the quantity and quality of crops (Abad et al., 2009). The annual global crop loss of plant-parasitic nematodes including root-knot nematodes estimated around eighty billion USD (Jones et al., 2013). However, the full extent of worldwide nematode damage is likely to be underestimated since growers are often unaware of their presence because the symptoms caused in the plants are often non-specific, making it difficult to attribute crop losses to nematode damage (Siddique and Grundler, 2018).

In Iran, two species, namely, *M. incognita* (Kofold and White, 1919) Chitwood, 1949 and *M. javanica* (Trub, 1885) Chitwood, 1949, have been frequently detected from major pistachio-producing regions of Kerman, Yazd, Isfahan, and Semnan provinces (Madani et al., 2012). The two species have been formerly reported in pistachio roots in California (McKenry and Kretsch, 1984).

Given the high economic impact of parasitic nematodes, numerous strategies have been developed for nematode control in agriculture. However, the application of chemical nematicides results in intensive environmental hazards (Zhang et al., 2017). Another way of controlling root-knot nematodes is the use of biocontrol agents. The effect of some probiotic bacteria as biocontrol agents of *M. incognita* on pistachio has been investigated, which has been successful in this field (Khatamidoost et al., 2015; Zeynadini-Risheh et al., 2018). Biocontrol using these agents also have some disadvantages: (1) biological control agents can be unstable; (2) they can only reduce the number of pathogens but cannot completely remove a pest; (3) controlling using these agents is a slow process; and (4) much planning and investment is required for developing a successful system. Therefore, using resistant cultivars is one of the most appropriate strategies for management of root-knot nematodes in pistachio orchards. Studying the host–nematode interactions will help find resistant rootstock for usage in breeding programs.

Several types of host–nematode relationships have been studied, such as carrot-*M. javanica*, soybean-pepper, cowpea and chili pepper-*M. incognita*, grapevine and peanut-*M. arenaria*, rice-*M. graminicola*, and coffee-*M. paranaensis* (Huang, 1986; Niblack et al., 1986; Bleve-Zacheo et al., 1998; Anwar and McKenry, 2000; Bendezu and Starr, 2003; Das et al., 2008; Moon et al., 2010; Cabasan et al., 2012; Alves et al., 2019). In some cases, no differences were found in nematode penetration to the susceptible and resistant cultivars. For example, *M. arenaria* in myrobalan plum (Voisin et al., 1999) and *M. incognita* in cowpea (Das et al., 2008) and cotton (Mota et al., 2013; Lopes et al., 2020). In some studies, the penetration of J2 was less in resistant plants than in susceptible plants like *M. arenaria* in grapevine (Anwar and McKenry, 2000), *M. incognita* in chili pepper (Moon et al., 2010), *M. graminicola* in rice (Cabasan et al., 2012), and *M. paranaensis* in coffee (Alves et al., 2019). J2 may penetrate resistant plants, but their development may be delayed, as reported in resistant maize and cotton genotypes-*M. incognita*, soybean-*M. arenaria*, potato-*M. fallax*, and rice-*M. graminicola* (Windham and Williams, 1994; Pedrosa et al., 1996; Kouassi et al., 2004; Faske and Starr, 2009; Cabasan et al., 2012). After penetration, the reaction leading to the death of the nematode, preventing the formation and development of feeding sites, is the hypersensitive response (HR) in resistant cultivars. It consists of localized and programmed cell death, resulting from recognition of pathogen *Avr* gene products by specific plant R proteins. This reaction is followed by plant defense responses such as reactive oxygen species production, activation of the salicylic acid pathway, jasmonic acid (Du et al., 2020), and production of phenolic compounds (Hung and Rohde, 1973; Bajaj and Mahajan, 1977; Pegard et al., 2005). HR has been observed in some cultivars infected by *Meloidogyne* species (Moon et al., 2010; Khallouk et al., 2011; Cabasan et al., 2014; Marini et al., 2016).

Madani et al. (2012) evaluated the egg mass and gall indices (EMI and GI) in Pistachio-*M. incognita* pathosystem in order to investigate the resistance between some pistachio rootstocks. In this study, *Pistacia terebinthus* from Australia and *P. atlantica* subsp. *mutica* (Fisch. & C.A.Mey.) Rech.f. rootstocks from Geno of Iran had the highest resistance, and Badami was regarded as the most susceptible rootstock, but the defense responses of pistachio in pistachio–*M. javanica* pathosystem have not been investigated yet.

The objectives of the present study were to (i) evaluate penetration, development, and reproduction of *M. javanica* on three domestic pistachio cultivars (Badami, Ghazvini, and Sarakhs) and a wild pistachio rootstock, Baneh; (ii) compare the resistance of the aforementioned rootstocks; and (iii) evaluate the resistance responses of the examined pistachio rootstocks.

## Materials and Methods

### Nematode inoculum

Several infested soil samples and roots having *Meloidogyne* galls were collected from pistachio gardens in Kerman and Khorasan Razavi provinces, Iran. All root-knot nematode populations were cultured on *Solanum lycopersicum* L. cv. Early Urbana. *Meloidogyne javanica* populations were identified based on female perineal pattern (Jepson, 1987) and species-specific primers javF/javR (Zijlstra et al., 2000). The most aggressive population was selected for the present study based on the GI and gall size, and then was reproduced on tomato roots for 4 months. The egg masses were picked up from the surface of the galled roots, and the eggs were extracted from the egg masses using 0.5% NaOCl in a blender (Hussey and Barker, 1973). The freshly hatched J2 were collected using a modified Baermann funnel and were counted under a stereomicroscope.

### Pistachio rootstocks

Seeds of Badami, Ghazvini, and Sarakhs rootstocks (*Pistacia vera* L.) and the wild pistachio, Baneh (*Pistacia atlantica*), were obtained from the Pistachio Research Center, Horticultural Sciences Research Institute, Agricultural Research, Education and Extension Organization (AREEO), Rafsanjan, Iran. These were soaked in 0.5% sodium hypochlorite for 3 min, washed with sterile distilled water, placed between two layers of wet cotton tissue in plastic bags, maintained at 4°C for one week and four weeks for selected pistachio cultivars and Baneh, respectively. The seeds were maintained at room temperature till germination. Germinated seeds were planted in plastic pots containing 1 kg of autoclaved soil and maintained under greenhouse conditions (25–30°C) with a 16-h light and 8-h dark regime.

### Resistance assays

The pistachio seedlings were inoculated with 3,000 J2 per plant (Pi = initial inoculum level) at the 8 to 10 leaf stage. The J2 were introduced into 3-cm-deep holes around the collar region of the plants. The inoculated pots were arranged in a complete randomized block design with six replications. They were irrigated and fertilized, as needed, and maintained under greenhouse conditions (25–30°C) with a 16-h light and 8-h dark regime. The pistachio seedlings were harvested at 120 dpi. The stem length and fresh root weight of pistachio seedlings, the nematode GI, EMI, and reproduction factor (RF) were measured. The roots were stained with Phloxine B and evaluated for GI or EMI, which had 1: 1 to 2 galls or egg masses; 2: 3 to 10 galls or egg masses; 3: 11 to 30 galls or egg masses; 4: 31 to 100 galls or egg masses; and 5: >100 galls or egg masses per root system (Hartman and Sasser, 1985). Eggs and juveniles were extracted from roots, as described by Hussey and Barker (1973), using 1% NaOCl solution and a blender, instead of manual agitation. The RF was estimated as RF = FP/IP, where FP is the final nematode population and IP is the initial nematode population (IP = 3,000 JV2s) (Oostenbrink, 1966).

Furthermore, the resistance index was measured according to Sasser et al. (1984) scale (Table 1).

**Table 1:** Resistance rating scale for *Meloidogyne javanica* based on the GI or EMI and RF (Sasser et al., 1984).

GI or EMI	RF	Resistance degree
1	<0.1	Highly resistant
2	£1	Resistant
3	>1	Moderately resistant
4	>1	Susceptible
5	>10	Highly susceptible

EMI, egg mass index; GI, gall index; RF, reproduction factor.

### Penetration and development assays

Ten seedlings of each pistachio rootstock were inoculated, as mentioned in resistance assays. This experiment was conducted with two replicates. J2 were introduced into 3-cm-deep holes around the collar region of the plant. Distilled water without nematodes was added to the control plants. Roots were removed from the pots and were carefully washed with tap water at 2 dpi, 4 dpi, 6 dpi, 10 dpi, 15 dpi, 21 dpi, 28 dpi, 35 dpi, and 45 dpi. The roots were stained with acid fuchsin to detect J2 penetration, their localization, and subsequent development within the roots. The roots were immersed in 3% NaOCl solution for 5 min, followed by rinsing with tap water to remove excess NaOCl (the root epidermis was dark, and for proper bleaching and preventing of damage to the cortex, the amount of NaOCl was increased, and the duration was reduced). The roots were then stained with 1 ml of 3.5% acid fuchsin stain (Byrd et al., 1983). The mixture was heated to the boiling level, then cooled at room temperature, and washed in running water. The roots were kept in acidified glycerin and were gently shaken for two days. After staining, root segments were observed under a stereomicroscope, and those parts showing nematode infection were mounted on a slide for observation under a light microscope (Olympus, BH-2).

### Statistical data analysis

Statistical analysis of the data obtained from different tests was performed using SPSS software (version 26), and the means were compared using Duncan’s multiple range test at a 95% confidence level.

## Results

### Comparison of *Meloidogyne javanica* effects on four pistachio rootstocks

#### Stem length

**Figure 1:**
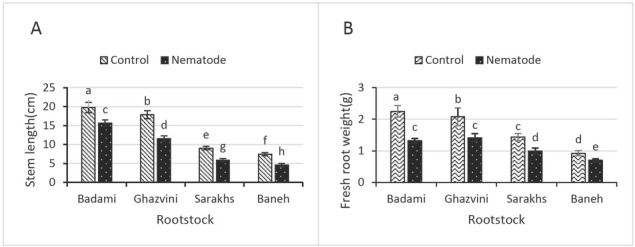
A- Effect of nematode infection on stem length of four pistachio rootstocks: Badami, Ghazvini, Sarakhs, and Baneh. B- Effect of nematode infection on fresh root weight of four pistachio rootstocks: Badami, Ghazvini, Sarakhs, and Baneh. The means with the same letter do not have significant differences according to Duncan’s multiple range test at the 95% confidence level.

The stem length of the four non-inoculated pistachio rootstocks was not the same, and, Badami, Ghazvini, Sarakhs, and Baneh ranked from longest to shortest, respectively. All the treated rootstocks had a significant stem length reduction at 120 dpi compared to their controls (Fig. 1A).

#### Fresh root weight

The fresh root weight differed between the tested plants. The highest fresh root weight in non-inoculated was found in Badami (2.25 g) and Ghazvini (2.07 g) rootstocks. Baneh had the least root weight in healthy controls (0.92 g). At 120 dpi, the fresh root weights of Badami, Ghazvini, Sarakhs, and Baneh rootstocks were 1.32 g, 1.41 g, 0.99 g, and 0.69 g, respectively. The reaction of them was not the same after infection with *M. javanica* in terms of fresh root weight; however, the root growth and development of all of the treated plants with nematode were reduced at 120 dpi compared to their controls (Fig. 1B).

#### Root-knot nematode resistance in pistachio rootstocks

The root-knot nematode resistance was evaluated based on the GI, EMI, and RF indices. The results of corresponding statistical analysis are shown in Table 2. Badami exhibited high levels of gall and egg mass indices (GI or EMI = 4), and the nematode had a high level of reproduction (RF average = 4.43), so it is ranked as the susceptible rootstock to *M. javanica*. By contrast, Baneh with GI or EMI = 2 and RF ≤ 1 (RF = 0.25) is classified as resistant rootstock. In Ghazvini and Sarakhs, RF was >1 (1.37 and 1.27, respectively), and GI or EMI was 3, so they were considered moderately resistant (MR) to *M. javanica* (Table 1).

**Table 2:** **Reaction of four pistachio rootstocks to *Meloidogyne javanica***.

Pistachio	EMI (g root)^-1^	GI (g root)^-1^	RF	Resistance
rootstock				
Badami	33.41^a^	64.18^a^	4.43^a^	S
Ghazvini	12.26^c^	29.98^b^	1.37^b^	MR
Sarakhs	16.49^b^	27.14^b^	1.27^b^	MR
Baneh	8.49^d^	17.23^c^	0.25^c^	R

EMI, egg mass index; GI, gall index; MR, moderately resistant; R, resistant; RF, reproduction factor; S, Susceptible. The means with the same letter do not have significant differences according to Duncan’s multiple range test at the 95% confidence level.

#### Penetration

**Figure 2:**
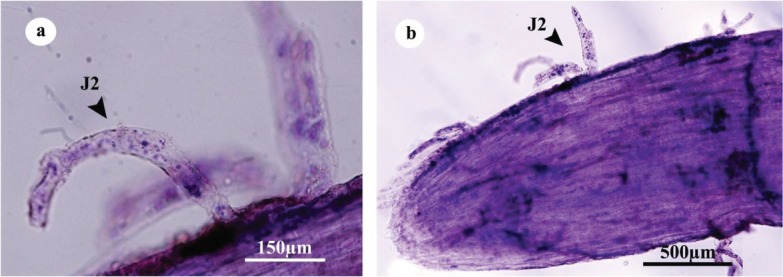
a,b: Penetration of J2 of *Meloidogyne javanica* in the root tip of pistachio rootstock roots after staining with acid fuchsin.

**Figure 3:**
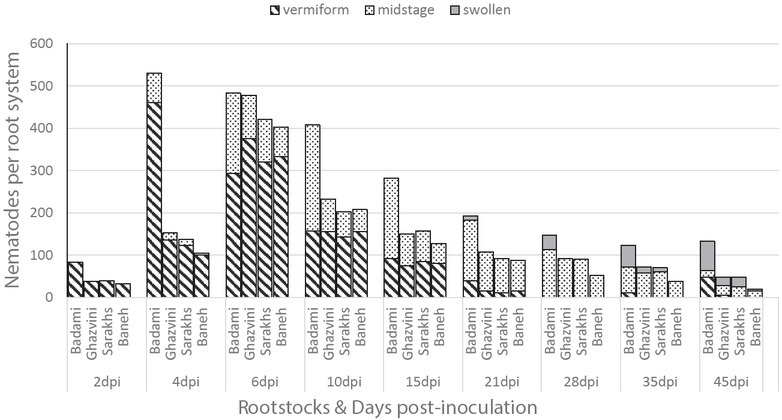
Dynamics of penetration and development of *Meloidogyne javanica* inside the roots of four pistachio rootstocks: Badami, Ghazvini, Sarakhs, and Baneh at 2 dpi, 4 dpi, 6 dpi, 10 dpi, 15 dpi, 21 dpi, 28 dpi, 35 dpi, and 45 dpi.

The results of acid fuchsin staining of the roots showed a high number of J2 were able to penetrate the root tip of all cultivars at 2 dpi (Figs. 2a,b). At 2 dpi and 4 dpi, the number of J2 that penetrated Badami rootstock was significantly higher than other rootstocks. At 4 dpi, Badami had the greatest penetration rate of the juveniles, but for Ghazvini, Sarakhs, and Baneh, the highest penetration rate was at 6 dpi. After 4 dpi, the number of penetrated J2 slightly decreased in Badami roots, but in Ghazvini, Sarakhs, and Baneh, it rapidly reduced after 6 dpi. Penetration rates of the J2 into Sarakhs and Baneh roots were lower than those in Badami and Ghazvini at 6 dpi. The least penetration rate was observed in Baneh (Fig. 3).

#### Development

**Figure 4:**
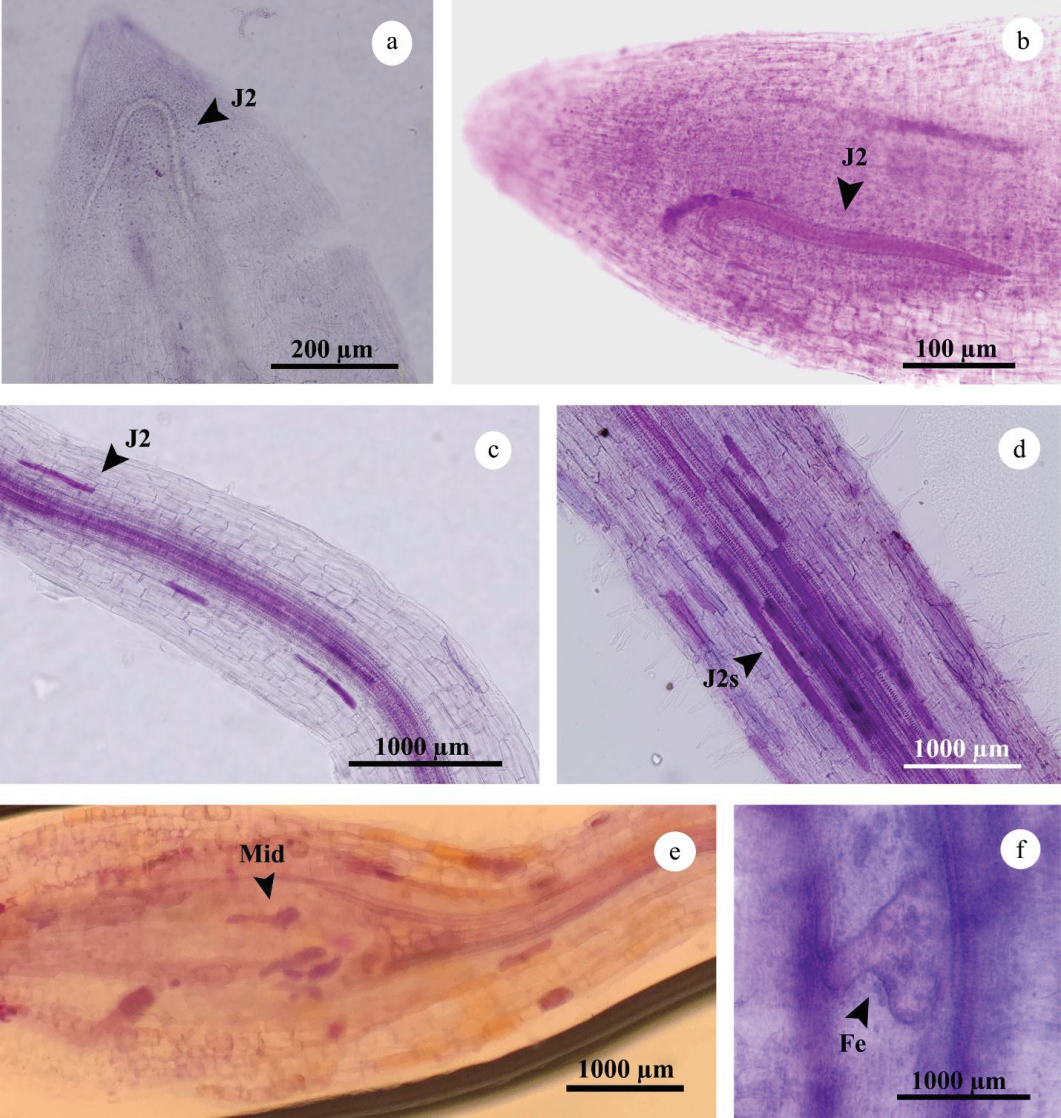
Development of *Meloidogyne javanica* in roots of pistachio rootstocks roots after staining with acid fuchsin. a,b: The J2 entering the vascular cylinder from the longitudinal growth area of the root; c,d: The J2 migrating in groups through the cortex; e: Midstages in vascular cylinder; f: female in vascular cylinder. Fe, female; J2, second-stage juvenile; Mid, third and fourth juveniles.

After penetration, the J2 immediately entered the vascular cylinder from the longitudinal growth area of the root (Figs. 4a,b) or migrated in groups through the cortex to reach thicker parts of the root (Figs. 4c,d). The cortex cells adjacent to the J2 were slightly swollen, but the giant cells did not form. The giant cells were only formed in the vascular cylinder and formed around the nematode head region. The first giant cells were observed in Badami at 4 dpi; for the other three rootstocks, they were observed at 6 dpi. The macroscopic symptom of root swelling was observed from 10 dpi onward. The first “midstage” or swollen juveniles (terminology after Windham and Wilhams, 1994) were observed at 4 dpi, but with lower extent in Ghazvini, Sarakhs, and Baneh cultivars (Figs. 3,4e). The first females were seen in Badami at 21 dpi, in Ghazvini and Sarakhs at 35 dpi, and in Baneh at 45 dpi (Figs. 3,4f). The first J2 from the new generation were obtained in Badami at 28 dpi and in Ghazvini at 35 dpi, but there were no J2 of the new generation seen in Sarakhs and Baneh at 45 dpi. So, there was more delay in nematode development in Baneh, Sarakhs, and Ghazvini, respectively, compared to that in Badami.

#### Resistance response of pistachio rootstocks to M. javanica

**Figure 5:**
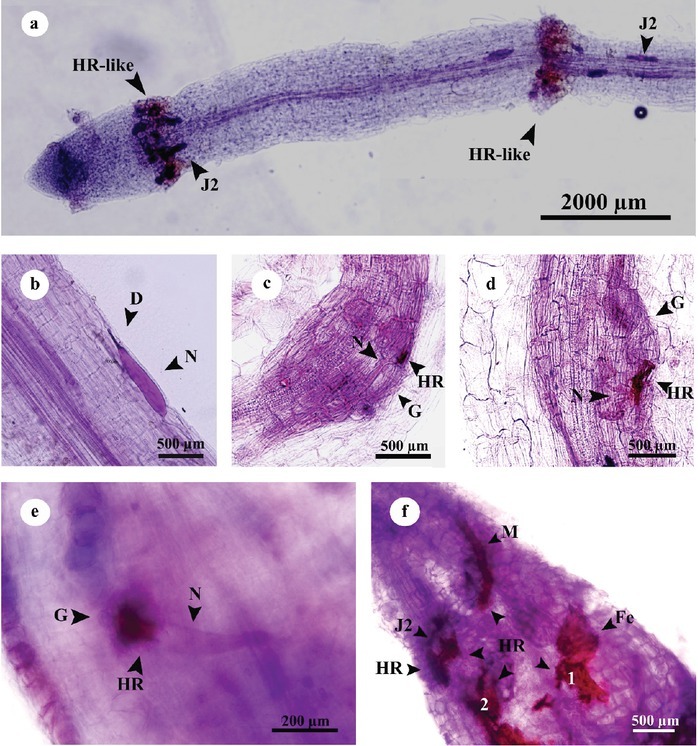
Resistance reaction of pistachio rootstocks roots to *M. javanica*, stained with acid fuchsin (cultivar Ghazvini); a: primary HR-like reaction led to the death of the J2, preventing them from progress; b: degeneration of J2 in the cortex; c–e: HR occurred in the vascular cylinder during the formation of feeding sites; f: degraded root tissue adjacent to the nematode feeding site due to HR (purple color) at 45 dpi. Male, female, and J2 could be seen simultaneously, and HR was activated around all of them. 1 and 2, two parts of the female torn due to cover slip pressure; D, degeneration; Fe, female; G, giant cell; HR, hypesnsetive respons; J2, second-stage juvenile; M, male; N, nematode.

An initial defense reaction was a hypersensitive response (HR)-like reaction observed in moderately resistant (Ghazvini and Sarakhs) and resistant (Baneh) rootstocks at 4 dpi and 6 dpi, respectively. It was characterized by necrosis of cells directly affected by nematode in the cortex. This HR-like reaction led to death of the J2 (Fig. 5a); however, some of the J2 died in the cortex without any HR response (Fig. 5b).

Another early defense response observed in moderately resistant (Ghazvini and Sarakhs) and resistant (Baneh) rootstocks at 10 dpi and 6 dpi, was programmed cell death (HR), which occurred in the vascular cylinder during the formation of feeding sites. It prevents development of nematodes and feeding sites and has led to nematode death (Figs. 5c–e).

Moreover, this reaction was more prevalent in Baneh than Ghazvini and Sarakhs. HR was not observed in Badami rootstock.

The late defense reaction observed in all of the rootstocks was malformation and destruction of the giant cells occurred from 15 dpi onward. It was characterized by browning of feeding sites close to the nematode head, indicating necrosis of tissues. Also, no alive nematodes were found in these galls (Fig. 5f).

## Discussion

During the present study, the reaction of three rootstocks and one wild-type pistachio was assessed against the infection by *Meloidogyne javanica* for the first time in Iran. Shoot and root biomass have already been used to assess the reaction of rootstocks (Stirling et al., 2011). Bhuiyan and Garlick (2021) stated in sugarcane plants infected with *M. javanica*, if plants with similar root biomass have different number of nematodes, then the difference is most likely due to different levels of resistance, but if a plant has smaller root system, the lower number of nematodes could be due to the limitations in the root biomass. So, it may be more desirable to compare shoot and root biomass of inoculated and uninoculated plants of the same rootstocks to assess the resistance level. This would allow a direct measure of the effect of the nematode on shoot and root growth. In the present study, the stem length and root development were not the same; therefore, the stem length and fresh root weight of each nematode-infected pistachio rootstocks are only relevant when compared to their control. The stem length and fresh root weight of all pistachio rootstocks infected with *Meloidogyne javanica* were decreased when compared to their respective controls. However, it seems the resistance in studied pistachio rootstocks is not because of different root biomass as fresh root weight in Badami is more than that in Ghazvini. The penetration of J2 in examined rootstocks is the same and again, the fresh root weight in Sarakhs is more than that in Baneh. General reduction of plant growth factors as the result of the infection by the root-knot nematode has already been documented. The reason for plant growth reduction is the absorbance of water and nutrients by root-knot nematodes (Karssen and Moens, 2006). Baneh, the most resistant cultivar, had the least weight loss. The negative correlation between fresh root weight reduction and resistance in pea cultivars against *M. incognita* (Sharma et al., 2006) and in rice against *M. graminicola* (Galeng-Lawilao et al., 2018) has already documented.

According to Roberts (2002), resistance of plants against nematodes refers to the ability of the plants to inhibit the development or reproduction of nematodes. The GI and EMI are considered good guides to evaluate resistance via the measurement of nematode establishment and reproduction in the host, respectively (Gomes et al., 2015). Soares et al. (2018) urged that the RF is a reliable factor in selecting resistant genotypes because it considers the final and initial populations of the nematode. The aforementioned indices are reliable standards for resistance assessments against root-knot nematode infection and have been previously applied in screening for *Meloidogyne* spp. resistance of various crops (Mota et al., 2013; Gomes et al., 2015; Lima et al., 2015; Soares et al., 2018; Brito et al., 2020). In the present study, the GI, EMI, and RF indices were used as measures to evaluate the resistance of pistachio rootstocks against *M. javanica*. The differences in GI, EMI, and RF indices between the four examined rootstocks showed their different levels of resistance against *M. javanica*. Based on these factors, Badami, Ghazvini, Sarakhs, and Baneh had the high, median, median, and low indices and ranked as susceptible, moderately resistant, moderately resistant, and resistant against the nematode, respectively.

In the previous and only available relevant study by Madani et al. (2012) using pistachio–*M. incognita* pathosystem, *Pistacia terebinthus* from Australia and *P. atlantica* rootstocks from Geno of Iran had the highest resistance, and Badami was regarded as the most susceptible, based on GI and EMI indices.

Mechanisms of resistance to root-knot nematodes can be categorized into pre- and post-infection steps (Anwar and McKenry, 2002; Bendezu and Starr, 2003). The pre-infection resistance is defined by the failure of nematode to penetrate the root (Bendezu and Starr, 2003; Pegard et al., 2005). During the post-infection resistance, the penetrated juveniles fail to develop to females (Anwar and McKenry, 2002). During this study, the J2 of *M. javanica* could penetrate to the roots of all four examined rootstocks. However, the penetration rates of J2 in Baneh (resistant) and Sarakhs (relatively resistant) were less than those in Badami (susceptible) and Ghazvini (relatively resistant) at 6 dpi. Although the same penetration rate of J2 in different cultivars of host plant was observed in Myrobalan plum-*M. arenaria* (Voisin et al., 1999), cowpea-*M. incognita* (Das et al., 2008), and cotton-*M. incognita* reaction (Mota et al., 2012; Lopes et al., 2020), in some other studies, resistant plants yielded in low penetration rates (Pegard et al., 2005; Proite et al., 2008).

After 4 dpi, the J2 number slightly decreased in Badami, but in Ghazvini, Sarakhs, and Baneh, it was rapidly declined after 6 dpi. Voisin et al. (1999) showed some of penetrated J2 into the resistant cultivars of myrobalan plum, emigrated from the root, and entered other host plants. The juvenile death in response to host defense responses or their emigration has already documented in the case of the resistant cultivars (Cabasan et al., 2012; Phan et al., 2018). The dead juveniles in the cortex were observed during the present study as well. Therefore, the more reduction of J2 in Baneh, Sarakhs, and Ghazvini than that in Badami rootstock showed there are resistance sources in them.

The first females were seen in Badami at 21 dpi, in Ghazvini and Sarakhs at 35 dpi, and in Baneh at 45 dpi. So, the nematode life cycle was delayed in Ghazvini and Sarakhs (relatively resistant) and Baneh (resistant). The delay in the life cycle of root-knot nematodes was observed in the resistant genotype of *Arachis* spp. (Proite et al., 2008). The inhibition of the development of *M. graminicola* was observed in resistant rice genotypes as well (Cabasan et al., 2012; Ji et al., 2015; Phan et al., 2018).

One of post-infection mechanisms of resistance is HR, a type of programmed cell death that is induced after the invasion of avirulent pathogens to prevent the spread of biotrophic pathogens (Huysmans et al., 2017). It has been observed at three different phases of plant parasitic nematode (PPN) infection in resistant plants: (1) in the cortex and epidermis during penetration and migration, (2) in vascular tissues during the initiation of feeding cell formation, and (3) in cells adjacent to developing feeding cells (Sato et al., 2019). The first phase of HR (herein named HR-like reaction), which occurred in the cortex and vascular cylinder at 4 dpi and 6 dpi was observed in Ghazvini, Sarakhs, and Baneh (moderately resistant and resistant), but not in Badami (susceptible). This reaction, which prevents the migration of J2 in the root, has already been observed in resistant cultivars of coffee (Lima et al., 2015), pepper (Pegard et al., 2005), and peanut (Proite et al., 2008) against root-knot nematode infection. Pegard et al. (2005) characterized this HR-like reaction displaying blue autofluorescence under UV light, proving the accumulation of phenolic compounds. This early HR reaction (in the epidermis and root cortex) expedites the success of the plant to overcome the nematode invasion (Bleve-Zacheo et al., 1998; Castagnone-Sereno et al., 2001). Some of J2 were however able to escape the initial defense reaction. In such cases, the secondary HR reaction occurred in the vascular cylinder, where they tried to form initial feeding cites. The secondary HR aims to prevent the formation of giant cells. During this phase, necrosis occurred in cells opposite to the nematode head. This reaction was mostly observed in Ghazvini, Sarakhs, and Baneh and, to a less extent, in Badami, at 6 dpi to 10 dpi, and has been previously reported in tomato at 1 dpi (Williamson, 1999), in pepper at 1 dpi to 3 dpi (Pegard et al., 2005), in soybean at 2 dpi to 3 dpi (Kaplan et al., 1979), and in coffee at 4 dpi to 6 dpi (Anthony et al., 2005). The third and the last HR, which was the last defense reaction against *M. javanica*, occurred in all pistachio rootstock at 15 dpi onward in the present study and inhibited the development of feeding cells by forming a barrier between feeding cells and surrounding cells to block nutrition and water supply, yielding in the giant cell collapse. Based on Cabasan et al. (2014), the late HR was the most common mechanism of resistance observed in all *Oryza glaberrima*-resistant genotypes. Similarly, coffee genotypes resistant to *M. exigua* exhibited early and late HR, the latter in case of the J2 escaped from the first defense reaction (Anthony et al., 2005). Lopes et al. (2020), who investigated the resistance reactions in *Gossypium* spp. against *M. incognita*, reported all observed/discussed three types of HR responses in pistachio rootstocks and urged that the early HR reaction was the most common mechanism. The prevalence of the primary HR reaction has already documented (Williamson and Kumar, 2006; Proite et al., 2008)

The expression of HR-like reaction and early and late HR defense responses in infected roots could indicate that multiple genes are likely to be involved in the resistance of pistachio rootstocks. The two latter HR reactions triggered in susceptible Badami rootstock as well, but in a slow rate and to a less extent, compared to those in moderately resistant and resistant rootstocks. However, none of studied pistachio rootstock were completely resistant to *Meloidogyne javanica*, so some of the penetrated J2 could reach the next generation, and the nematode inoculum remained in the soil. Therefore, the use of moderately resistant and resistant cultivars could be included in combination with other measures in an integrated root-knot nematode management. Desired features of moderately resistant or resistant rootstocks against *M. javanica*, as revealed herein, may be used in pistachio-breeding programs in future.

## Conclusion

During the present study, both host pistachio plants and root-knot nematode-related indices were assessed in greenhouse experiments, and as the result, the Badami cultivar was proven to be susceptible, two cultivars Ghazvini and Sarakhs were proven as relatively resistant, and the wild pistachio, Baneh, was proven to be resistant against *M. javanica*. The three latter rootstocks could be used in breeding programs of this economic crop.
